# Neural Hierarchy of Color Categorization: From Prototype Encoding to Boundary Encoding

**DOI:** 10.3389/fnins.2021.679627

**Published:** 2021-07-19

**Authors:** Mengdan Sun, Luming Hu, Xiaoyang Xin, Xuemin Zhang

**Affiliations:** ^1^Center for Psychological Sciences, Zhejiang University, Hangzhou, China; ^2^Beijing Key Laboratory of Applied Experimental Psychology, Faculty of Psychology, National Demonstration Center for Experimental Psychology Education, Beijing Normal University, Beijing, China; ^3^State Key Laboratory of Cognitive Neuroscience and Learning, IDG/McGovern Institute for Brain Research, Beijing Normal University, Beijing, China

**Keywords:** color perception, categorization, fMRI, frontal, categorical structure, visual cortex

## Abstract

A long-standing debate exists on how our brain assigns the fine-grained perceptual representation of color into discrete color categories. Recent functional magnetic resonance imaging (fMRI) studies have identified several regions as the candidate loci of color categorization, including the visual cortex, language-related areas, and non-language-related frontal regions, but the evidence is mixed. Distinct from most studies that emphasized the representational differences between color categories, the current study focused on the variability among members within a category (e.g., category prototypes and boundaries) to reveal category encoding in the brain. We compared and modeled brain activities evoked by color stimuli with varying distances from the category boundary in an active categorization task. The frontal areas, including the inferior and middle frontal gyri, medial superior frontal cortices, and insular cortices, showed larger responses for colors near the category boundary than those far from the boundary. In addition, the visual cortex encodes both within-category variability and cross-category differences. The left V1 in the calcarine showed greater responses to colors at the category center than to those far from the boundary, and the bilateral V4 showed enhanced responses for colors at the category center as well as colors around the boundary. The additional representational similarity analyses (RSA) revealed that the bilateral insulae and V4a carried information about cross-category differences, as cross-category colors exhibited larger dissimilarities in brain patterns than within-category colors. Our study suggested a hierarchically organized network in the human brain during active color categorization, with frontal (both lateral and medial) areas supporting domain-general decisional processes and the visual cortex encoding category structure and differences, likely due to top-down modulation.

## Introduction

Humans can distinguish among thousands of hues but use only a small number of color categories. How does our brain assign the fine-grained perceptual representation of color into discrete color categories? This question relates to the classic debate on the interaction between language and perception. Linguistic relativists claim that color categories are shaped by the language we speak, while universalists postulate that color categories are independent of language and formed based on perceptual mechanisms (for review, see Regier and Kay, 2009; Siuda-Krzywicka et al., 2019a; Witzel, 2019).

Centering on this debate, recent functional magnetic resonance imaging (fMRI) studies have investigated the neural basis of color categorization in the human brain, but the results remain inconsistent (Siok et al., 2009; Brouwer and Heeger, 2013; Bird et al., 2014; Persichetti et al., 2015; Liu et al., 2019; Siuda-Krzywicka et al., 2019b). Several brain regions have been identified as the candidate loci of color categorization in the adult human brain, including the visual cortex (e.g., V4 and VO1; Persichetti et al., 2015), language-related areas (Siok et al., 2009), and non-language-related frontal regions (Bird et al., 2014; Persichetti et al., 2015). Siok et al. (2009) found that, for color stimuli presented in the right visual field, the activity involved in discriminating different-category colors was much greater in the left hemisphere language regions and visual areas V2/V3 than the activity involved in discriminating same-category colors. Siok et al. (2009) proposed that the language areas might serve as a top-down control source that interacts with and modulates the activity of V2/3. The evidence of top-down modulation on color perception was more direct in Brouwer and Heeger (2013), who found that within-category colors showed greater similarity of blood oxygen level-dependent (BOLD) response patterns in V4 and VO1 than colors from different categories when participants actively categorized color stimuli but not when they passively viewed the stimuli. Despite the absence of an effect of color categories on adaptation effects in the visual cortex, Persichetti et al. (2015) observed increased direct BOLD responses to stimuli near the category boundary in hV4 during passive viewing and in all visual areas during active categorization.

While these aforementioned studies focused on how color categorization connects with color perception and/or language, a few recent studies have provided an alternative possibility that color categorization could be distinct from both perception and language (Bird et al., 2014; Liu et al., 2019; Siuda-Krzywicka et al., 2019b). Bird et al. (2014) reported a larger BOLD response for cross-category compared with within-category sequential color changes in the middle frontal gyrus (MFG), which supports a domain-general role in categorization. Similar to Bird et al. (2014) and Liu et al. (2019) found that colors are encoded categorically at high levels of the cognitive hierarchy, including the MFG. In addition, the intraparietal sulcus (IPS) and presupplementary motor area (SMA) played a role in making decisions based on the perceptual and categorical color information. Persichetti et al. (2015) also revealed several language-independent frontal regions that encode color categories. More surprisingly, Siuda-Krzywicka et al. (2019b) reported the robustness of color categorization in a patient with impaired color naming. These results allow us to look beyond the classical perception–language debate and to rethink color categorization from a domain-general perspective (Siuda-Krzywicka et al., 2019b).

However, most of the previous studies emphasized the representational differences in different color categories, the information the brain contains to discriminate between categories, to reveal category encoding in the brain. Specifically, their analyses were based on the assumption that two simultaneously or successively presented cross-category colors should evoke greater differences in BOLD amplitudes or in multivariate patterns than two within-category colors. Although encoding for differences between categories is one key element of category representations, the variability between members within a category, referred to as the internal structure of the category (Rosch, 1973; Rosch and Mervis, 1975; Davis and Poldrack, 2014), is also crucial. Regarding the structure of the color category, different color exemplars vary in the representativeness for each category. Focal colors or category prototypes are maximally representative for each category, and boundary colors are minimally representative (Heider, 1972; Abbott et al., 2016; Witzel, 2019). Actually, the representational differences in different category exemplars have been addressed in both studies of color and other domains (Swaminathan and Freedman, 2012; Brouwer and Heeger, 2013; Bae et al., 2015; Persichetti et al., 2015; Seger et al., 2015; Braunlich et al., 2017). Brouwer and Heeger (2013) proposed a model of categorical clustering, claiming that neural color space in some visual areas is transformed during color categorization and that neurons tuned to a color near the center of a color category gained larger increases in activity than neurons tuned to boundary colors. Persichetti et al. (2015) observed increased responses to stimuli near the category boundary in the visual cortex. We must admit that the previous studies (Bird et al., 2014; Persichetti et al., 2015; Liu et al., 2019) using the adaptation paradigm included different exemplars within a color category, which were able to capture the within-category variability (if it was present). However, a reasonable conclusion is that the within-category variability was not systematically manipulated and examined in previous studies of color categorization and that the neural evidence of encoding for category structure is still lacking. Studies of categorization in other domains, on the other hand, have provided more evidence regarding how neural activities covary with distances from category boundaries and/or category prototypes during visual categorization (Grinband et al., 2006; Swaminathan and Freedman, 2012; Davis and Poldrack, 2014; Roy et al., 2014; Seger et al., 2015; Braunlich et al., 2017). For instance, the frontoparietal network is responsible for making a categorical decision and is sensitive to the distance from category boundaries (Grinband et al., 2006; Swaminathan and Freedman, 2012; Roy et al., 2014; Seger et al., 2015; Braunlich et al., 2017), and sensory regions (e.g., occipitotemporal cortex) represent category typicality and thus are sensitive to the distance from category prototypes (Davis and Poldrack, 2014; Braunlich et al., 2017).

In this study, we intended to probe the neural basis of color categorization by assessing whether the activities of some brain regions covary with the degree of the representativeness of color stimuli for each category. Specifically, the current study compared and modeled brain activities evoked by color stimuli with varying distances from the category boundary in an active categorization task. We hypothesized that color-category-selective regions would either track a boundary model or a prototype model. The boundary model predicts greater neural responses to colors around the category boundary and lower responses with increasing distances from the boundary, and the prototype model predicts greater neural responses to prototype colors and lower responses with increasing distances from category prototypes. We expected to identity functionally distinct networks involving both low-level and high-level processes during color categorization (Grinband et al., 2006; Swaminathan and Freedman, 2012; Roy et al., 2014; Seger et al., 2015; Braunlich et al., 2017).

## Materials and Methods

### Participants

Seventeen healthy right-handed undergraduate and graduate students with normal or corrected-to-normal vision were recruited from Beijing Normal University. All participants reported normal color vision. None of them had any neurological or psychological disorders. All participants provided written informed consent before participating in the study and received payment. The study was approved by the Institutional Review Board of the National Key Laboratory of Cognitive Neuroscience and Learning, School of Brain and Cognitive Sciences at Beijing Normal University.

### Stimuli

**FIGURE 1:**
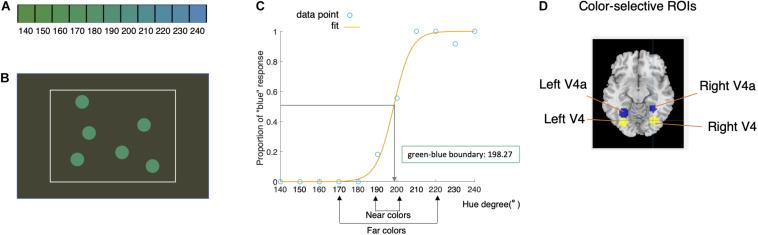
Experimental design and data analyses. **(A)** Eleven hues used in the task: 140–240° at a step size of 10° in the CIELAB color space. **(B)** Six disks (0.5° radius) with an identical surface hue were displayed at random positions inside a rectangular area (14.4°× 9.58°, white frame) with a gray background for a duration of 1 s. **(C)** Behavioral responses of a typical participant. Each data point represents the proportion of “blue” responses for each color, which were fitted with a psychometric function to determine the individual green–blue boundary. After the green–blue boundary was defined (198.27° in this case), the near colors were defined as the two colors nearest to the individual green–blue boundary. The far colors included one green color and one blue color, with the green far color two-step (i.e., 20°) greener than the green near color and the blue far color two-step bluer than the blue near color. **(D)** Four color-selective ROIs, namely, the left V4, right V4, left V4a, and right V4a, were defined as spheres with a radius of 9 mm [116 voxels; peak voxel: (−30 −73 −11), (31 −70 −11), (−29 −55 −13), and (29 −47 −17), respectively].

Eleven color stimuli were sampled from the green–blue region in the CIELAB space (*L*^∗^ = 70, a radius of 29), which only varied in hue (140–240°, step size: 10°, Figure 1A). During scanning, the stimuli were projected onto a monitor placed at the back of the magnet bore from a video projector. The monitor was calibrated using a Minolta Konica CS-150 colorimeter.^1^ The *xyY* values of the white point (*x* = 0.4106, *y* = 0.4464, *Y* = 49.74) and the monitor primaries (R: *x* = 0.6342, *y* = 0.3457, *Y* = 8.66; G: *x* = 0.3895, *y* = 0.5740, *Y* = 25.40; B: *x* = 0.1805, *y* = 0.1353, *Y* = 2.41) were measured. The input–output value of each channel was also measured to define the gamma curve. This information was used to determine the appropriate RGB values for each color stimulus as suggested by Brainard et al. (2002). Participants viewed the monitor screen through a mirror (of 48 cm × 36 cm) at a distance of approximately 110.5 cm from their eyes. The size of the display was 24.5° × 18.4°.

### Procedure

We adopted an event-related design. Six disks (0.5° radius) with an identical surface hue were displayed at random positions inside a rectangular area (14.4° × 9.58°) with a gray background for a duration of 1 s (Figure 1B). The hues of the color stimuli were sampled in a randomized order. Interstimulus intervals ranged from 2 to 5 s, in steps of 1.5 s. Participants were required to indicate whether the surface color of the disk was green or blue by pressing a button with the index finger or the middle finger. The response window was within 1.5 s after the stimulus onset, and responses generated after that time were not recorded. The experiment included two runs in total. Each run included 12 conditions (11 colors and a blank presentation), and each condition included six trials in each run. A 1-min rest was provided between the two runs.

### Image Acquisition

Scanning was performed in a 3-T MRI scanner (Siemens Magnetom Trio, A Tim System, Siemens, Malvern, PA, United States). A standard 12-channel head coil was used to ensure coverage of the entire brain. High-resolution T1-weighted MPRAGE anatomical images (TR = 2,300 ms, TE = 2.86 ms, flip angle = 9°, 144 slices, matrix size = 256 × 256, voxel size = 1 mm × 1 mm × 1.33 mm) were acquired for each participant before the tracking task. Functional images were acquired with a gradient-echo single-shot echo-planar sequence (TR = 2,000 ms, TE = 30 ms, flip angle = 90°, 33 slices, interleaved slice order, matrix size = 64 × 64, FOV = 200 mm × 200 mm, voxel size = 3.125 mm × 3.125 mm × 4.025 mm).

### Data Analysis

#### Definition of Near and Far Colors

We firstly defined *near colors* (i.e., colors near the green–blue boundary) and *far colors* (i.e., colors far from the green–blue boundary) for each participant based on their individual green–blue boundary for further analyses to investigate whether the brain activity was sensitive to distance from the color boundary. The individual green–blue boundary was determined using behavioral responses. For each participant, the proportion of “blue” responses was fitted with a psychometric function defined as 1/(1 + exp (− (x−α)/β), where α is the threshold (estimated value at which “blue” would be reported half of the time), namely, the green–blue boundary in our study (Figure 1C). The behavioral green–blue boundaries of the participants ranged from 189.49° to 214.57° (mean = 200.74°, SD = 6.70°).

For each participant, the near colors were defined as the nearest two colors to the individual green–blue boundary (one color on each side), resulting in one green near color and one blue far color (Figure 1C). The far colors included one green color and one blue color, with the green far color two steps (i.e., 20°) greener than the green near color and the blue far color two steps bluer than the blue near color. For instance, if the boundary of a participant was 198.27°, then the near colors would be 190° [green near color) and 200° (blue near color), and the far colors would be 170° (green far color)] and 220° (blue far color, Figure 1C). The near–far distance (20°) was chosen so that the far colors would not be as so close to the near colors, and at the same time, the far colors were not out of our color sample range (considering the participant with a boundary of 214.57°).

#### Behavioral Analyses

Reaction times (RTs) during categorization were analyzed to assess the effect of distance from the color boundary. We regarded RTs in trials with responses out of the response window (i.e., longer than 1.5 s after stimulus onset) as 1,500 ms. This practice might mask the actual differences between conditions but would not cause false positives. RTs for four color stimuli (two near colors and two far colors) were analyzed. A two (distance: far, near) × two (category: green, blue) repeated-measure ANOVA was then carried out on mean RTs.

#### Neuroimaging Analyses

Preprocessing and statistical analyses of fMRI data were performed using SPM12 (Wellcome Department of Imaging Neuroscience, University College London, United Kingdom^2^). Preprocessing of the functional data, including slicing timing, realignment, coregistration, spatial normalization to Montreal Neurological Institute (MNI) space using unified segmentation method, and spatial smoothing with a 6-mm full-width at half-maximum Gaussian kernel, was performed for each run. A high-pass filter cutoff was set to 128 s.

For each participant, the evoked BOLD responses to 11 color stimuli were modeled using a general linear model in which the time series was convolved with the canonical hemodynamic response function (HRF) with its temporal and dispersion derivatives (Friston et al., 1999; Ward et al., 2008; Brouwer and Heeger, 2013). The derivatives were included because the HRF of an individual voxel may have differed from the canonical HRF. The variance of the estimated response amplitudes across runs was smaller with the derivatives included than without them (Brouwer and Heeger, 2013). The values obtained for the derivative regressors were not included in further analyses, and we only reported analyses related to amplitudes of the HRF. Nuisance regressors consisted of six head motion parameters and a constant regressor for each run.

##### Contrasts

For the group-level analyses, we compared the neural responses to the near colors to the responses to far colors to assess the effect of distance from the color boundary on brain activity. We obtained the contrast maps (near > far and far > near) for each participant and analyzed them in a second-level random-effects model. Group statistics were computed for each contrast to examine areas of activation for the group as a whole. The clusterwise threshold was set to control the familywise error (FWE) rate at *p* < 0.05. A primary voxel-level threshold was set as *p* < 0.001 to define clusters (cluster size > 20).

##### Model Fit

We extracted *t*-values reflecting the effect of each color stimulus on each voxel in regions showing near–far effects and fitted von Mises functions (Jammalamadaka and Sengupta, 2001; Brouwer and Heeger, 2013; Bae et al., 2015) to these normalized *t*-values to validate the distance effects (near > far or far > near) revealed in the contrast analyses indexing the boundary model (i.e., neural response covaried with distance from category boundary) or prototype model (i.e., response covaried with distance from category prototype), rather than any biased local effects: $f\,\left(x\right)=\frac{\exp\left(k\times \left[\cos\left(x-\mu \right)\right]\right)}{\left[2\times \pi \times I_{0}\,\left(k\right)\times m\right]}+b$. The von Mises distribution is considered as the circular analog of the normal distribution and thus was used because the color space is circular. In this model, the value of *x* corresponds to the location of the color stimuli in color space (i.e., hue degree). This function included four free parameters: μ (mean parameter), *k* (standard deviation parameter), *m* (modulation depth), and *b* (baseline *t*-value). The mean parameter μ determines the location of the peak of the curve; thus, it indicates the value of the color stimulus that evoked the greatest neural response (i.e., preferred color value). The mean parameter μ is the parameter of interest in our study.

If a brain region tracked to the boundary model, then the best μ estimate for this region should be the value of category boundaries. If a brain region tracked to the prototype model, then the best μ estimate should be the value of category prototypes. As each color category (i.e., green and blue) has its unique prototypes, we separately fitted the model to the green and blue color stimuli. For the green category, the model was fitted for each participant to the hues from the 140° hue to the second-nearest hue that was bluer than the participant’s subjective green–blue boundaries. For instance, if a participant’s green–blue boundary is 205°, then the green color samples input into the model for this participant would range from 140° to 220°. Using this approach, responses to color stimuli around the individual green–blue boundary were input into the model, and it would produce an accurate estimate (μ) of the color boundary if a brain region truly tracked to the boundary model. Similarly, for the blue category, the model was fitted for each participant to the hues from the 240° hue to the second-nearest hue that was greener than the green–blue boundary of the individual. Non-linear least squares fitting was performed to estimate parameters for each brain region (voxels were pooled together), each color category, and each participant. Parameters were initialized to multiple starting values in an attempt to avoid local maxima.

##### Regression

In the contrast analyses described above, we compared distantly positioned color pairs (near vs. far) to reveal the distance effects, which possibly ignored the information carried in the shapes of individual psychophysics curves. We then conducted subject-level simple linear regression analyses to investigate how individuals’ brain activities covary with their differential behavioral responses during categorization based on the psychophysics curves. Specifically, the *t*-values reflecting the effects of hue 200° and hue 210° for each participant were obtained as dependent variables, and the absolute hue distances between the hues and the individual green–blue boundary were computed as a regressor. For a certain hue (e.g., 200°), the varying hue distance from the category boundary reflects individual differences in category membership. The reason why we focused on the hue 200° and hue 210° is that, as the two nearest color stimuli around the group green–blue boundary (200.74°), the brain responses should reflect the greatest individual differences in color categorization. The data for hue 200° and hue 210° were pooled together (*N* = 34).

##### Regions of Interest

Structural regions of interest (ROIs) were defined based on a previous study (Purmann and Pollmann, 2015) that reported the MNI coordinates of peak voxels of four color-selective areas. According to this study, four ROIs (Figure 1D), namely, the left V4, right V4, left V4a, and right V4a, were defined as spheres with a radius of 9 mm [116 voxels; peak voxel: (−30 −73 −11), (31 −70 −11), (−29 −55 −13), and (29 −47 −17), respectively]. The percent signal change for the near and far colors in each ROI was obtained for each participant using the MarsBaR toolbox (Brett et al., 2002). For each ROI, a two (distance: far, near) × two (category: green, blue) repeated-measure ANOVA was then carried out on the percent signal change. For ROIs exhibiting distance effects, we extracted *t*-values reflecting the effect of each color stimulus for each voxel, fitted von Mises functions to these normalized *t*-values, and estimated the preferred color (μ) for each color category and each participant, as in the whole-brain analyses (see the details in section “Model Fit” listed above).

In addition, we conducted a brain–behavior correlation analysis between the percent signal change and the distance from the individual green–blue boundary using the hue samples 200° and 210° to examine whether the four ROIs tracked individual differences in color categorization. Considering the non-independence of the data for hue 200° and hue 210°, they were averaged for each participant prior to performing the correlation (*N* = 17).

## Results

### Behavioral Results

**FIGURE 2:**
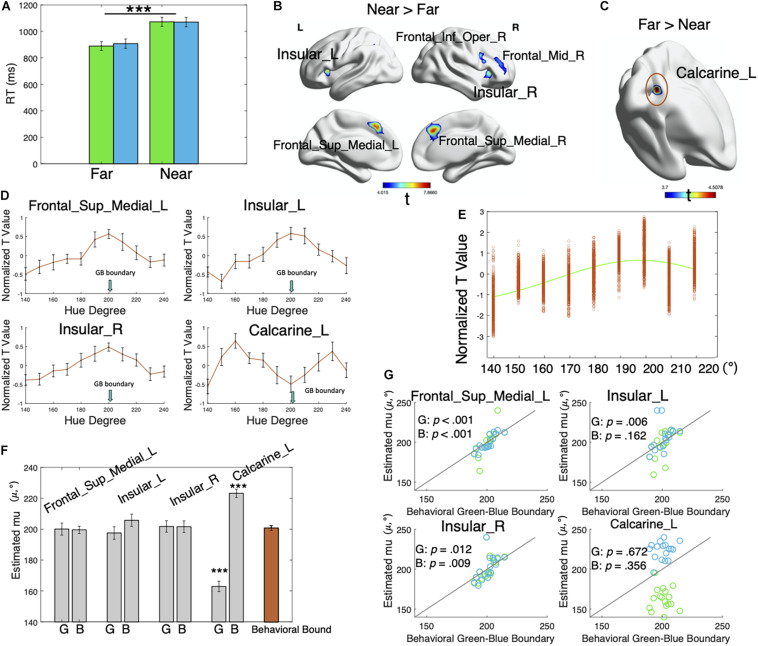
Behavioral and fMRI results. **(A)** Average RTs for the far and near colors. Green bars represent the green far/near colors, and blue bars represent the blue far/near colors. Error bars represent ± SEM. The RTs were significantly different for the near colors and the far colors (*p* < 0.001). Neither the effect of category nor the interaction was significant (*p* > 00.05). ***p < 0.001. **(B)** Regions showing greater activation for the near colors than the far colors. The clusterwise threshold was set to control the familywise error (FWE) rate at *p* < 0.05. A primary voxel-level threshold was set as *p* < 0.001 to define clusters. Cluster size > 20. **(C)** Regions showing greater activation in response to the far colors than the near colors. Voxel-level *p* < 0.001, cluster-level uncorrected *p* = 0.053. **(D)** Mean voxel-averaged normalized *t*-values of 17 participants in the four regions showing distance effects (near > far and far > near). Error bars represent ± SEM. **(E)** Normalized *t*-values for green color stimuli in the Frontal_Sup_Medial_L region of a typical participant. Each data point represents the voxel-level *t*-value for each green color, which was then fitted with a von Mises function to determine the preferred color direction of the region. **(F)** Mean estimated μ (indicating preferred color values, gray bars) of 17 participants for each brain region and each color category. “G” represents the green category, and “B” represents the blue category. The orange bar represents the behavioral green–blue boundaries. Error bars show ± SEM. The estimates (μ) in the Calcarine_L significantly differed from the behavioral green–blue boundaries (*p* > 0.001). ***p < 0.001. **(G)** Correlation between individual estimated μ (indicating preferred color direction) for each brain region and each color category and behavioral green–blue boundaries. Green dots represent the green category, and blue dots represent the blue category. Lines in the plots show the function *y* = *x*. Dots close to the lines indicate a convergence of estimated μ with behavioral green–blue boundaries.

The two (distance: far, near) × two (category: green, blue) repeated-measure ANOVA of RTs revealed a significant effect of distance [*F*(1, 16) = 107.99, *p* < 0.001, partial η^2^ = 0.871]. As expected, the RTs for near trials were longer than those for far trials (Figure 2A), suggesting larger uncertainty in the categorization of boundary colors. Neither the effect of category nor the interaction was significant [category: *F*(1, 16) = 0.15, *p* = 0.704, partial η^2^ = 0.009; interaction: *F*(1, 16) = 0.225, *p* = 0.642, partial η^2^ = 0.014].

### Neuroimaging Results

#### Near vs. Far

As shown in Figure 2B and Table 1, the near colors induced greater activities in the lateral fronto-insular regions and medial prefrontal cortex than the far colors [the clusterwise threshold was set to control the FWE rate at *p* < 0.05; a primary voxel-level threshold was set to *p* < 0.001 to define clusters (cluster size > 20)], including the inferior and middle frontal gyri in the right hemisphere, bilateral insula, and medial parts of the bilateral superior frontal cortices.

**TABLE 1:** Coordinates of activation peaks in the contrasts (near > far and far > near) and regression analyses.

**Brain region**		**Cluster size**	***T***	**MNI coordinates**		
				***x***	***y***	***z***
Threshold: voxel-level *p* < 0.001, cluster-level FWE-corrected *p* < 0.05						
	**Near > far**					
Region 1	Frontal_Sup_Medial_L	499	7.87	0	33	42
	Frontal_Sup_Medial_R		7.51	9	33	39
	Cingulate_Mid_R		4.85	12	21	30
Region 2	Insular_L	98	7.07	−33	18	3
Region 3	Insular_R	771	6.46	33	27	0
	Frontal_Inf_Oper_R		5.81	36	9	30
			5.65	51	15	21
Threshold: voxel-level *p* < 0.001, cluster-level uncorrected *p* = 0.053						
	**Far > near**					
Region 4	Calcarine_L	30	4.84	−12	−60	18
Threshold: voxel-level *p* < 0.001, cluster-level FWE-corrected *p* < 0.05						
	**Negative correlation**					
	Frontal_Sup_Medial_L	145	4.51	0	21	42
	Frontal_Inf_Oper_R	115	4.44	48	15	0
	Insular_R		4.41	39	15	−3
	Frontal_Inf_Oper_R		4.24	48	6	15

No brain regions showed significantly larger activities in the far trials than in the near trials using the aforementioned threshold. When we applied no cluster-level correction, a cluster of voxels (voxel-level *p* < 0.001, cluster-level uncorrected *p* = 0.053) in the left calcarine sulcus exhibited greater responses to the far colors than the near colors (Figure 2C and Table 1).

#### Prototype vs. Boundary Model

We extracted normalized *t*-values reflecting the effect of each color stimulus on each voxel in regions showing near–far effects (Table 1): Frontal_Sup_Medial_L (region 1), Insular_L (region 2), Insular_R (region 3), and Calcarine_L (region 4). Figure 2D shows the mean voxel-averaged normalized *t*-values of 17 participants in these four regions. Notably, the normalized *t*-value (signed) indicates the magnitude of the effect of the color stimuli on neural responses. For example, a value of -1 represents a small effect, and a value of 1 represents a large effect. It appeared that brain regions, including the Frontal_Sup_Medial_L, Insular_L, and Insular_R, were the most sensitive to colors around the boundary and that the sensitivity decreased for colors away from the boundary (mean behavioral green–blue boundary: 200.74°). In contrast, in the left calcarine, the effects of stimuli were the lowest for the boundary colors and increased with the increasing distance from the color boundary. More importantly, the stimulus effects decreased again after a certain point on both sides. The activities in the left calcarine appeared to be associated with the distance from category prototypes, while the other three regions were associated with the distance from the category boundary.

We fitted the normalized voxel-level *t*-values with Mises functions to quantitatively explore whether these brain regions tracked to the boundary model (i.e., neural response covaried with distance from category boundaries) or prototype model (i.e., response covaried with distance from category prototypes) (Figure 2E). The estimated μ values (indicating the preferred color direction) of the participants for each brain region and each color category are plotted in Figures 2F,G. It showed that the estimates (μ) in the Frontal_Sup_Medial_L, Insular_L, and Insular_R converged with the behavioral green–blue boundaries of the individual for both green and blue categories, while the estimates in the Calcarine_L diverged from them. We then conducted paired *t*-tests and correlation analyses, separately for each color category and each brain region to examine the consistency between the estimates (μ) and the behavioral green–blue boundaries at the group level. No significant differences were observed between these pairs (all *p*s > 0.05, Figure 2F; see the detailed statistics in Supplementary Table 1) except for the pairs for the Calcarine_L region [green: *t*(16) = 9.721, *p* < 0.001, Cohen’s *d* = 2.358; blue: *t*(16) = −7.971, *p* < 0.001, Cohen’s *d* = −1.933]. The correlation analyses between the behavioral green–blue boundaries and the estimates (μ) for each color category and each brain region showed that all of these pairs were significantly correlated (all *p*s < 0.05, Figure 2G; see the detailed statistics in Supplementary Table 2) except for the two pairs in the Calcarine_L [green: *r* = −0.111, *p* = 0.671, CI = (−0.562, 0.390); blue: *r* = −0.239, *p* = 0.356, CI = (−0.273, 0.645)] and one pair in the Insular_L [blue: *r* = 0.355, *p* = 0.162, CI = (−0.152, 0.714)].

These results suggested that the responses of the Frontal_Sup_Medial_L, Insular_L, and Insular_R to color stimuli covaried with the distance from the category boundary because the estimated preferred color directions (μ) in these regions were mostly consistent with the behavioral green–blue boundaries, according to the *t*-tests and correlation analyses. In contrast, the estimated preferred color directions (μ) in the Calcarine_L region were independent of the category boundaries. Instead, for the green color stimuli, the responses in the Calcarine_L covaried with the distance from the hue with a group mean of 162.94°; for the blue stimuli, the responses covaried with the distance from the hue with a mean degree of 223.20° (Figure 2F). Most likely, the Calcarine_L region represented category prototypes rather than category boundaries.

#### Regression

**FIGURE 3:**
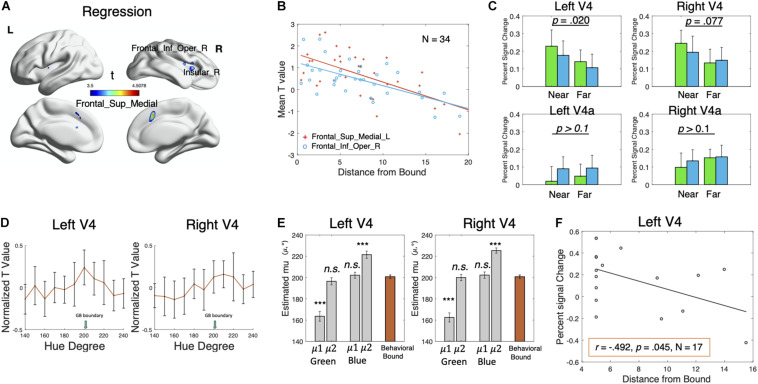
Results of the regression and ROI analyses. **(A)** Regions showing a negative correlation between brain activation and distance from category bound for hue 200° and hue 210° (pooling together) at the individual level. The clusterwise threshold was set to control the familywise error (FWE) rate at *p* < 0.05. A primary voxel-level threshold was set to *p* < 0.001 to define clusters. Cluster size > 20. **(B)** Scatterplot showing the linear relationship correlation between the mean *t*-value in the Frontal_Sup_Medial_L and Frontal_Inf_Oper_R and individual distance from category bound for hue 200° and hue 210° (pooled together). **(C)** Average percent signal change for the far and near colors in four ROIs: left V4, right V4, left V4a, and right V4a. Green bars represent the green far/near colors, and blue bars represent the blue far/near colors. Error bars represent ± SEM. The percent signal change was (marginally) significantly different between the near colors and the far colors in the left V4 (*p* = 0.02) and right V4 (*p* = 0.077). **(D)** Mean voxel-averaged normalized *t*-values of 17 participants in the left V4 and right V4 ROIs showing distance effects (near > far). Error bars represent ± SEM. **(E)** Mean estimated μ1 and μ2 (indicating preferred color values, gray bars) of 17 participants for each color category in the left V4 and right V4 ROIs after fitting the new model incorporating the two mean parameters. The orange bar represents the behavioral green–blue boundaries. Error bars represent ± SEM. ***p < 0.001. **(F)** Scatterplot showing the linear relationship correlation between percent signal change in the left V4 and individual distance from the category bound for hue 200° and hue 210° (averaged).

For the hue 200° and 210°, we observed a negative correlation between the distance from the individual green–blue boundary and the brain activities in the Frontal_Sup_Medial, Frontal_Inf_Oper_R, and Insular_R (Figures 3A,B and Table 1), similar to the results we obtained in the contrast analyses. This result suggested that the activation in these fronto-insular areas increases with the decreasing distances from the category boundary at the individual level. In addition, a small cluster in the left lingual gyrus showed a weaker negative correlation (voxel-level *p* < 0.001, cluster-level uncorrected *p* = 0.036), suggesting that the color-selective sensory areas may also encode boundary colors. No regions showed significant positive correlation.

#### ROI Analyses

The two (distance: far, near) × two (category: green, blue) repeated-measure ANOVA of the percent signal change for each ROI showed a significant effect of distance in the left V4 [*F*(1, 16) = 6.71, *p* = 0.020, partial η^2^ 0.296] and marginally significant in the right V4 [*F*(1, 16) = 3.58, *p* = 0.077, partial η^2^ = 0.183], as the near colors produced larger responses than the far colors (Figure 3C). The distance effect was not significant in either the left or right V4a (*p*s > 0.1). The effect of the category or interaction was not significant in any of the four ROIs (*p*s > 0.1). Thus, the V4 areas, especially the left V4, were potentially tracking to the boundary model. To examine this possibility, we extracted normalized *t*-values reflecting the effect of each color stimulus on each voxel in the left and right V4 (Figure 3D), fitted the normalized voxel-level *t*-values with Mises functions, and estimated μ for each participant, each ROI, and each color category. For the left V4 ROI (Supplementary Figures 1A,B), the estimates for the green and blue categories were significantly different from the behavioral green–blue boundary [green: *t*(16) = −2.61, *p* = 0.019, Cohen’s *d* = −0.632; blue: *t*(16) = 2.53, *p* = 0.022, Cohen’s *d* = 0.613]. For the right V4 ROI, the estimates for the green category were significantly different from the behavioral green–blue boundary [*t*(16) = −2.51, *p* = 0.023, Cohen’s *d* = −0.609] but not for the blue category [*t*(16) = −1.35, *p* = 0.197, Cohen’s *d* = −0.326]. The correlation analyses between the behavioral green–blue boundaries and the estimates (μ) showed that none of these pairs were significantly correlated (*p*s > 0.1). The results of the *t*-tests and the correlation analyses suggested inconsistency between the estimates (μ) and the behavioral green–blue boundaries in the V4 ROIs. The activity in the V4 area might not be closely associated with the behavioral category boundaries, although it did show a difference between the near and far color stimuli.

Alternatively, we suspected that the V4 area tracked to both the boundary model and the prototype model. To test this possibility, we fitted the normalized voxel-level *t*-values with a new model which is the sum of two Von Mises functions: $f\,\left(x\right)=\frac{\exp\left(k\times \left[\cos\left(x-\mu \,1\right)\right]\right)}{\left[2\times \pi \times I_{0}\,\left(k\right)\times m\right]}+\frac{\exp\left(k\times \left[\cos\left(x-\mu \,2\right)\right]\right)}{\left[2\times \pi \times I_{0}\,\left(k\right)\times m\right]}+b$. The new distribution is clustered around two mean values: μ1 and μ2. If the V4 area truly tracked to both the boundary model and the prototype model, the new model with two mean parameters (μ1 and μ2) should outperform the old model with one mean parameter (μ). Moreover, the estimated μ1 and μ2 should correspond to the values of category prototypes and category boundaries, or vice versa. Within a category, we designated the smaller mean parameter as μ1 and the larger mean parameter as μ2. For simplicity, we assumed that these two von Mises functions in the new model have the same standard deviation (characterized by *k*) and modulation depth (characterized by *m*).

We used adjusted *r*^2^ as an indicator of goodness of fit to avoid the problem of overfitting and to compare the performance between the new model and the old model. For each ROI (left and right V4) and each color category, the adjusted *r*^2^ values explained by the two models for the 17 participants were compared using paired *t*-tests, showing that the new model was better than the old model for the green and blue categories in each ROI (*p*s < 0.01, see the detailed statistics in Supplementary Table 3). This result was in favor of a “mixed model” for the bilateral V4 region, which encodes both category prototypes and boundaries, rather than a pure boundary model. We then conducted paired *t*-tests to examine the consistency between the estimates (μ1 and μ2) and the behavioral green–blue boundaries at the group level (Figure 3E and Supplementary Table 4). For both V4 ROIs, the estimates μ1 (mean: 163.6° in the left V4 and 162.5° in the right V4)] of the green category were significantly different from the behavioral category boundaries [left V4: *t*(16) = −6.87, *p* < 0.001, Cohen’s *d* = −1.666; right V4: *t*(16) = −8.04, *p* < 0.001, Cohen’s *d* = −1.949], while the estimates μ2 (mean: 196.5° in the left V4 and 200.00° in the right V4) were not [left V4: *t*(16) = −1.20, *p* = 0.249, Cohen’s *d* = −0.290; right V4: *t*(16) = −0.30, *p* = 0.770, Cohen’s *d* = −0.072]. The estimates μ2 (mean: 221.5° in the left V4 and 225.3° in the right V4) of the blue category were significantly different from the behavioral category boundary [left V4: *t*(16) = 5.83, *p* < 0.001, Cohen’s *d* = 1.413; right V4: *t*(16) = 9.81, *p* < 0.001, Cohen’s *d* = 2.379], while the estimates μ1 (mean: 202.2° in the left V4 and 202.3° in the right V4°) were not [left V4: *t*(16) = 0.48, *p* = 0.636, Cohen’s *d* = 0.117; right V4: *t*(16) = 0.52, *p* = 0.612, Cohen’s *d* = 0.125]. Regarding the correlation (Supplementary Figure 1C), only the estimates μ2 for the green category in the right V4 ROI were significantly correlated with the behavioral category boundary [*r* = 0.553, *p* = 0.021, CI = (0.099, 0.817)]. All the other pairs were not significantly correlated (*p*s > 0.1). In summary, at the group level, the estimated boundaries predicted by the model (μ2 for the green category and μ1 for the blue category) were not significantly different from the individual behavioral category boundaries in the V4 ROIs. However, based on the correlation analyses, they were less predictive of the individual behavioral boundaries than the fronto-insular areas. In addition, the estimated category prototypes (μ1 for the green and μ2 for the blue) were similar to the estimates in the left calcarine.

Finally, we observed a negative correlation between the distance from the individual green–blue boundary and the percent signal change in the left V4 ROI [*r* = −0.492, *p* = 0.045, CI = (−0.786, −0.014); Figure 3F]. No significant correlation was observed in the right V4, left V4a, or right V4a (*p*s > 0.1). These findings further confirmed the hypothesis that the left V4 ROI encodes boundary colors.

### Supplementary Analyses

#### Validation of Distance Effects

In our primary contrast analyses, the near colors were defined as the nearest two colors to the individual green–blue boundary (one color on each side). Here, we used the second closest pair to the category boundary as the near pair in order to include more data in our analyses, referred to as near2 colors (see the detailed analyses in the Supplementary Materials). The near2 > far effects were similar to but weaker than the results of near > far comparison in the primary analyses (Supplementary Table 5 and Figure 2A). The right MFG showed greater responses in the near2 trials than in the far trials, and the medial superior frontal gyri (SFGmed) were also included when a looser threshold was used. This finding is reasonable because the near2 colors were closer to the far colors than the near colors. The far > near2 comparison did not show significant effects.

#### Representational Similarity Analysis

The representational similarity analysis (RSA; Kriegeskorte et al., 2008) has been extensively adopted in the literature to examine categorical representation. While our study focused on the within-category variability, we additionally used the RSA approach to assess the representation of cross-category differences in the brain. Undoubtedly, the near colors in our study were accompanied by greater task difficulty and decisional uncertainty in categorization than the far colors. Thus, larger activities on the near trials in the frontal areas are not necessarily due to encoding of category boundaries but due to performing general decision-making functions. The RSA approach has the advantage of controlling these confounding factors and examines whether the regions showing distance effects carry information of categorical differences. We first conducted RSA on the regions showing distance effects in the primary contrast analyses: Frontal_Sup_Medial_L (region 1), Insular_L (region 2), Insular_R (region 3), and Calcarine_L (region 4), using ROI-based procedure (see the detailed analyses in the Supplementary Materials). We correlated the neural representational dissimilarity matrix (RDM) for each region (Supplementary Figure 2C) with a predefined category model (Supplementary Figure 2B) and found a significant association between the predefined category model and the neural RDM for Insular_L (*r* = 0.280, *p* = 0.029) and Insular_R (*r* = 0.311, *p* = 0.026) but not Frontal_Sup_Medial_L (*r* = 0.076, *p* = 0.293) and Calcarine_L (*r* = 0.103, *p* = 0.205).

In addition, we conducted RSA on the four predefined ROIs in the visual cortex (left and right V4, left and right V4a). Interestingly, we found that neural RDM in the bilateral V4a showed significant association with the predefined category model (left V4a: *r* = 0.296, *p* = 0.015; right V4a: *r* = 0.357, *p* = 0.007), while no significant correlation was found in the bilateral V4 (left V4: *r* = −0.117, *p* = 0.795; right V4: *r* = −0.079, *p* = 0.714). These results suggested that both the bilateral insular cortices and sensory areas (bilateral V4a) carry the information of categorical differences of color. Moreover, the categorical differences and the within-category variability (revealed by previous amplitude analyses) are encoded in different areas of the visual cortex.

## Discussion

The current study investigated how the neural responses to color stimuli covary with the distance from the category boundary in order to reveal the neural basis of color categorization. We identified a hierarchically organized network in the human brain, with the bilateral fronto-insular areas and the medial superior frontal gyri sensitive to colors around the category boundary and the visual cortex sensitive to both category prototypes and boundaries. In addition, the bilateral fronto-insular cortex and the V4a showed greater pattern similarity for within-category colors than cross-category colors. We suggest that the prefrontal regions are likely to support domain-general decisional processes and that the fronto-insular cortex encodes the outcome of color categorization. Regarding the categorical representation in the sensory areas, both the early visual cortex and V4 encode internal category structures of color, while the V4a encodes categorical differences. The categorical representation in visual areas likely results from top-down modulation during active color categorization.

### Color Categorization in Frontal Regions

It was found that color stimuli near the individual green–blue boundary produced increasing activities in the bilateral medial superior frontal cortices and bilateral insulae extending to the pars opercularis of the inferior frontal gyri (IFG). The enhanced activity for boundary colors in these frontal areas might result from category-boundary representation, or instead, decisional processes, as color stimuli around the category boundary were confounded with greater categorization difficulty and/or attention investment in our study. According to previous evidence, the fronto-insular cortex and the medial frontal areas are part of the salience network (Seeley et al., 2007; Menon and Uddin, 2010; Ham et al., 2013; Otti et al., 2013). The salience network was known to be associated with high-level functions, such as initiation of cognitive control (Menon and Uddin, 2010) and error and conflict processing (Klein et al., 2007; Ham et al., 2013). Using shape stimuli, Seger et al. (2015) reported similar regions (i.e., bilateral anterior insulae and medial frontal cortex) showing greater activity for near-bound stimuli during categorization, which was interpreted as greater conflict than those far from the decisional bound. In a subsequent study, Braunlich et al. (2017) documented an association between decisional uncertainty in categorization and activity in regions of the saliency network using a less strict threshold. In our study, the individual green–blue boundaries estimated by the model fit in the fronto-insular and medial frontal cortical regions perfectly converged with the behavioral category boundaries at the group level, suggesting that the activities in these regions are closely related to decisional processes. A reasonable conclusion is that the fronto-insular regions and medial frontal areas support decisional processes during color categorization. More interestingly, the representational similarity analyses revealed that the bilateral insulae carry category information, as cross-category colors exhibited larger dissimilarity in brain patterns than within-category colors. Considering that the insula has been consistently reported to be involved in perceptual decision-making, which is independent of stimulus modality (Deen et al., 2011; Chang et al., 2013; Lamichhane et al., 2016), the insula is unlikely to represent domain-specific color categories in our study. A more plausible explanation is that the insulae contain the categorical decision or categorization output from a sensory analysis, which subsequently guides behavior in choosing the appropriate response (Binder et al., 2004; Roy et al., 2014). In other words, we propose that the category information in the insular cortex is only decodable in a task that requires a response or a decision.

In addition to the bilateral fronto-insular cortex and medial SFGs, colors near the category boundaries evoked larger activation in the right MFG. The involvement of MFG in color categorization has been reported in several studies (Bird et al., 2014; Liu et al., 2019). According to Bird et al. (2014), the brain patterns in within-category blocks were more similar to the patterns in other within-category blocks than to those in cross-category blocks during passive viewing. Their study implicated the role of MFG in the categorical representation of color. However, using a similar adaptation paradigm, Persichetti et al. (2015) failed to reveal evidence of categorical processing in the MFG. Instead, they reported some separate frontal clusters showing categorical effects only during active color categorization. Liu et al. (2019) also identified greater activity in the bilateral MFG for cross-category colors than within-category colors, together with the insular cortex. However, their study differed from the study of Bird et al. (2014) because Liu et al. (2019) required explicit judgments of color categories. In summary, Bird et al. (2014) published the only study supporting the hypothesis that color is represented categorically in the MFG in the absence of overt judgment, while the involvement of the MFG or other lateral prefrontal regions in other studies was accompanied by high-level decisional factors (Persichetti et al., 2015; Liu et al., 2019). In addition, studies of categorical processing in other domains suggested that the MFG is associated with uncertainty in perceptual categorization (Hansen et al., 2011, 2012a,b). Moreover, the left and right MFG might play different roles, as the right MFG is involved in general decision-making, while the left MFG is related to perceptual uncertainty. According to this line of reasoning, we attributed the activation of the right MFG in our study to greater decisional uncertainty.

### Encoding Within-Category Variability and Cross-Category Differences in Visual Areas

Importantly, our study revealed that the information about both the category structure (i.e., within-category variability) and cross-category differences is carried in the visual areas during active categorization. Specifically, the left primary visual cortex (anterior calcarine region) showed larger responses for the color stimuli far from the boundary than the near-boundary colors at a loose threshold. The modeling results suggested that the responses to color stimuli in the left V1 covaried with the distance from focal colors within each category, indicating encoding of category prototypes. In contrast, the V4 areas showed more complex patterns when representing color categories. Within the V4 areas, colors near the category boundary produced increased activities compared with those far from it in ROI analyses, and a negative correlation was observed between the distance of color stimuli from the individual green–blue boundary and the percent signal change in the left V4 ROI. Based on these findings, the V4 cortex, particularly the left V4, is selective to category boundaries. Moreover, the results of modeling to V4 further revealed that the V4 encodes not only category boundaries but also category prototypes, as the data from the V4 were better explained by the model with two mean parameters than that with only one mean parameter for one category. Using RSA, we found that the bilateral V4a areas showed greater dissimilarity in brain pattern for cross-category than within-category colors but not the V4 areas or the V1 area. The results from pattern analyses and amplitude analyses seem to suggest that the internal structure or variability within a category and the between-category differences are represented in different ways and in distinctive visual areas. To our knowledge, this is the first study to reveal simultaneous representation of two different types of categorical color information in the visual cortex.

Whether or not the perceptual representation is shaped by color categories has been the focus of the language–perception debate (for review, see Regier and Kay, 2009; Siuda-Krzywicka et al., 2019a; Witzel, 2019). Compared with the view that categorical representation is inbuilt into the visual system, a recent and more plausible hypothesis is that fine-grained color representation is transformed into categorical encoding due to top-down modulation (Siok et al., 2009; Brouwer and Heeger, 2013), which may explain our findings. The boundary encoding in the V4 likely results from feedback from frontal regions, because the frontal regions showed more intense selectivity to boundary colors and greater predictivity of behavioral decision than V4. Furthermore, the novel findings of increased activities in V1 and V4 in response to focal colors were consistent with the categorical clustering model proposed by Brouwer and Heeger (2013) that neurons tuned to a color near the center of a color category gained larger increases than neurons tuned to boundary colors during transformation of the neural color space in visual areas due to top-down modulation. They also proposed that the source of this top-down modulation might be featural attention. However, our study did not obtain evidence of increased activities for category prototypes in frontal regions associated with attention. Instead, we observed a small cluster (size: 15 voxels) in the anterior part of the right superior and middle temporal gyrus (peak voxel: 54, −3, −25) that showed larger responses for far colors than near colors in the primary contrast analyses using a loose threshold (voxel-level *p* < 0.001, cluster-level uncorrected). This result may suggest that the enhanced responses to category prototypes in the visual areas are likely due to their closer associations with linguistic labels than other category exemplars (Siok et al., 2009). Therefore, our study suggested two different top-down sources that might be responsible for the representation of category prototypes and boundaries in the visual areas during active color categorization. Notably, with the absence of active color categorization, Persichetti et al. (2015) observed increased responses to stimuli near the category boundary in the visual areas, suggesting a possibility that within-category variability might be inherent in the visual system. The lack of a control experiment (i.e., passive viewing) prevents us from examining whether the categorical encoding in the visual areas is truly task-modulated. However, combined with the compelling evidence from the frontal networks, we postulate that the top-down modulation hypothesis is more reasonable.

Finally, as we mentioned above, a small cluster in the anterior part of the right superior and middle temporal gyrus showed larger responses to far colors than near colors using a loose threshold, suggesting weak distance effects on language-related areas during color categorization. Moreover, the categorical encoding in the visual cortex seemed more pronounced in the left hemisphere, suggesting the potential association between color perception and language. However, evidence has suggested that left lateralization is independent of language processing (Kosslyn et al., 1989; Holmes and Wolff, 2012); thus, the lateralization observed in our study cannot guarantee the involvement of language. Overall, the evidence for language processing was weak in our study, in favor of the dissociation of color categorization and color naming (Siuda-Krzywicka et al., 2019b).

### Specificity and Generality of Color in Visual Categorization

On the one hand, the domain of color has furnished a locus of the language–perception debate due to its relation to language. Investigations of the neural mechanism underlying color categorization have mainly focused on color-selective visual areas and language regions from a domain-specific perspective (for a review, see Siuda-Krzywicka et al., 2019a). On the other hand, studies of perceptual categorization in other domains used different low-level features (e.g., shape and motion) and probed how the brain transforms fine-grained visual features into arbitrary categories from a domain-general perspective (for a review, see Freedman and Assad, 2016). Our study suggested that the neural hierarchy involved in color categorization includes distinctive networks, with sensory areas encoding specific category information and frontal regions determining a decisional bound between two categories. These findings are comparable to the findings from the categorization research in other domains (for a review, see Freedman and Assad, 2016). However, color categorization has its specificity, as it is associated with learned language; we are “trained” to categorize color in daily life, and it might have a biological basis. This specificity may result in more tangled networks underlying color categorization. For instance, our study found a weak distance effect (far > near) in language-related areas, suggesting that language processing might be responsible for the enhanced responses to category prototypes in the visual areas. Accordingly, our study suggested that both generality and specificity of color categorization with categorization in other domains must be considered.

## Data Availability Statement

The raw data supporting the conclusions of this article will be made available by the authors, without undue reservation.

## Ethics Statement

The studies involving human participants were reviewed and approved by Institutional Review Board of the National Key Laboratory of Cognitive Neuroscience and Learning, School of Brain and Cognitive Sciences, Beijing Normal University. The patients/participants provided their written informed consent to participate in this study.

## Author Contributions

MS: conceptualization, methodology, data collection and analysis, and writing. LH: data collection and writing-reviewing. XX: data analysis. XZ: conceptualization, supervision, and funding acquisition. All authors contributed to the article and approved the submitted version.

## Conflict of Interest

The authors declare that the research was conducted in the absence of any commercial or financial relationships that could be construed as a potential conflict of interest.

## Footnotes

1 https://sensing.konicaminolta.us

2 http://www.fil.ion.ucl.ac.uk/spm
