# Supplementary material for: Neural Hierarchy of Color Categorization: From Prototype Encoding to Boundary Encoding
Source: Front Neurosci. 2021 Jul 19;15:679627. doi: 10.3389/fnins.2021.679627 (PMC8327959; doi:10.3389/fnins.2021.679627)
Supplement: Supplementary file 1 [file Data_Sheet_1.docx]

# Supplementary Material

**Supplementary analyses**

*Redefinition of Near colors*

In our primary contrast analyses, the Near colors were defined as the two colors nearest to the individual green-blue boundary (one color on each side). Here we used the second closest pair to the category boundary as the Near pair in order to include more data in our analyses, referred to as Near2 colors. For instance, if a participant’s boundary was 198.27°, then the Near2 colors would be 180° (green Near-color) and 210° (blue Near-color), and the Near2-Far distance would be 10° (Far colors would still be 170° and 220°).

*Representational similarity analyses*

We conducted representational similarity analyses (Kriegeskorte, Mur & Bandettini, 2008) on the regions showing distance effects as well as the ROI regions in the visual cortex using ROI-based procedure. These regions include Frontal_Sup_Medial_L, Insular_L, Insular_R, Calcarine_L, left V4, right V4, left V4a, and right V4a. The *t*-value images of 11 color stimuli were calculated to capture the activation patterns, which were generated from unsmoothed data. For each region, the activation patterns within a sphere (radium = 9 mm) centered at the peak voxel (Table 1) were extracted to calculate the neural representational dissimilarity matrix (RDM) using the CoSMoMVPA toolbox (Oosterhof, Connolly, & Haxby, 2016). A 11 $\times$ 11 neural RDM was obtained for each region and each participant, where the dissimilarity measure was computed by one minus Pearson correlation. The 17 participants’ neural RDMs were then averaged to obtain a group-level neural RDM.

For the RSA analysis of categorical representation, we compared the neural RDM with a predefined category model. For each participant, a 11 $\times$ 11 matrix representing the categorical differences between two colors was constructed. We first assigned the category membership (‘green’ or ‘blue’) based on individual’s behavioral green-blue boundary. Then in the individual category model, within-category pairs were assigned a value of 0, and cross-category pairs were assigned a value of 1. Then a group-level category model was computed by averaging the participants’ category models (Fig. S2B). We correlated this group-level category model with the actual group-level neural RDM for each region to assess whether the pattern of dissimilarity across color stimuli was captured by our specific model. For both the category model and the neural RDM for each region, the below diagonal matrix triangle was vectorized to form a dissimilarity vector with 55 entries, on which Pearson correlation coefficient was computed. The significance of correlation was assessed by permutation tests. For each participant, we randomly shuffled the category labels (“green” or “blue”) for each color stimulus, based on which individual permutated category model was obtained. All participants’ permutated 11 $\times$ 11 matrices were averaged to obtain a group-level permutated category model. We correlated this permutated category model with the neural RDM. This process was repeated 1,000 times to obtain a null distribution of correlation, separately for each region. The proportion of the null distribution greater than the measured value was designated as the (one-tailed) *p* value.

**Supplementary references**

Kriegeskorte, N., Mur, M., & Bandettini, P. (2008). Representational similarity analysis - connecting the branches of systems neuroscience. *Frontiers in systems neuroscience*, *2*, 4. https://doi.org/10.3389/neuro.06.004.2008

Oosterhof, N. N., Connolly, A. C., & Haxby, J. V. (2016). CoSMoMVPA: multi-modal multivariate pattern analysis of neuroimaging data in Matlab/GNU Octave. *Frontiers in neuroinformatics*, 10, 27.

**Supplementary tables**

Table S1 Statistics of estimated $\mu$ for each brain region and each color category (participant-averaged) and results of *t*-tests between the estimates versus the behavioral green-blue boundaries.

| Brain region | Green | | | | Blue | | | |
| --- | --- | --- | --- | --- | --- | --- | --- | --- |
|  | MEAN | SD | t | p | MEAN | SD | t | p |
| Frontal_Sup_Medial_L | 200.084 | 16.144 | 0.229 | 0.822 | 199.576 | 9.817 | 0.908 | 0.377 |
| Insular_L | 197.522 | 16.787 | 0.976 | 0.344 | 205.726 | 16.382 | -1.341 | 0.199 |
| Insular_R | 201.584 | 15.409 | -0.337 | 0.741 | 200.736 | 6.699 | -0.279 | 0.783 |
| Calcarine_L | 162.935 | 13.841 | 9.721 | <.001 | 223.199 | 11.227 | -7.971 | <.001 |

Table S2 Correlation between the estimates and the behavioral green-blue boundaries.

| Brain region | Green | | | |  | Blue |  |
| --- | --- | --- | --- | --- | --- | --- | --- |
|  | r | p | CI | | r | p | CI |
| Frontal_Sup_Medial_L | 0.773 | <.001 | | [0.465, 0.914] | 0.863 | <.001 | [0.653, 0.950] |
| Insular_L | 0.633 | 0.006 | | [0.219, 0.854] | 0.355 | 0.162 | [-0.152, 0.714] |
| Insular_R | 0.596 | 0.012 | | [0.162, 0.837] | 0.611 | 0.009 | [0.184, 0.844] |
| Calcarine_L | -0.111 | 0.671 | | [-0.562, 0.390] | 0.239 | 0.356 | [-0.273, 0.645] |

Table S3 Statistics of goodness of fit (adjusted r square, participant-averaged) explained by the new model incorporating two mean parameters ($\mu1$ and $\mu2$) and the old model incorparating one mean parameter ($\mu$) in V4 ROIs.

| Brain region | Green | | | |  | Blue |  |  |  |
| --- | --- | --- | --- | --- | --- | --- | --- | --- | --- |
|  | New | Old | t | p | | New | Old | t | p |
| Left V4 | 0.40$\pm0.$18 | 0.29$\pm0.16$ | 4.95 | <.001 | | 0.47$\pm0.16$ | 0.37$\pm0.18$ | 3.84 | .001 |
| Right V4 | 0.39$\pm0.15$ | 0.29$\pm0.13$ | 6.61 | <.001 | | 0.45$\pm0.15$ | 0.35$\pm0.18$ | 3.85 | .001 |

Table S4 Statistics of estimated $\mu1$ and $\mu2$ (participant-averaged) for each color category in V4 ROIs predicted by the new model incorporating two parameters.

| Brain region | Green | | Blue | | |
| --- | --- | --- | --- | --- | --- |
|  | $\mu1$ | $\mu2$ | | $\mu1$ | $\mu2$ |
| Left V4 | 163.60$\pm$19.17 | 196.48$\pm$14.16 | | 202.19$\pm$11.52 | 221.50$\pm$13.95 |
| Right V4 | 162.50$\pm$17.67 | 200.00$\pm$12.37 | | 202.30$\pm$12.34 | 225.34$\pm$10.09 |

Table S5 Coordinates of activation peaks in the contrast analysis (Near2 > Far)

|  |  | |  | |  | MNI coordinates | | | |
| --- | --- | --- | --- | --- | --- | --- | --- | --- | --- |
| Brain region | Cluster size | T | | x | | | y | z | |
| *Threshold: voxel-level p < 0.001, cluster-level FWE-corrected p < 0.05* | | | | | | | | |  |
| **Near2 > Far** |  |  | |  | | |  |  | |
| Frontal_Mid_2_R | 167 | 5.90 | | 51 | | | 42 | 15 | |
|  |  | 5.26 | | 48 | | | 39 | 24 | |
|  |  | 4.99 | | 57 | | | 15 | 39 | |
| *Threshold: voxel-level p < 0.001, cluster-level uncorrected p = 0.021* | | | | | | | | | |
| Frontal_Sup_Medial_R | 49 | 4.51 | | 3 | | | 33 | 45 | |

**Supplementary figures**

Fig. S1 Results of ROI analyses. (A) Mean estimated $\mu$ (indicating preferred color values, grey bars) of 17 participants for each color category in left V4 and right V4 ROIs by the old model incorporating one mean parameter. The orange bar represents the behavioral green-blue boundaries. Error bars represent $\pm$ SEM. (B) Correlation between the individual estimated $\mu$ (i.e., preferred color values) and the green-blue boundary for each color category in left and right V4 fitting with the old model incorporating one mean parameter. Green dots represent the green category, and blue dots represent the blue category. Lines in the plots show the function y = x. Dots close to the lines indicate a convergence of the estimates with the behavioral green-blue boundaries. (C) Correlation between the individual estimated $\mu1$ and $\mu2$ (i.e., preferred color values) and the behavioral green-blue boundaries for each color category in left and right V4 fitting with the new model incorporating two mean parameters. Lines in the plots represent the function y = x. Dots close to the lines indicate a convergence of the estimates with the behavioral green-blue boundaries.

Fig. S2 Results of supplementary analyses. (A) Regions showing greater activation for the Near2 colors than the Far colors. The cluster-wise threshold was set to control the family-wise error (FWE) rate at *p* < 0.05. A primary voxel-level threshold was set as *p* < 0.001 to define clusters. cluster size >20. (B) Predefined category model: larger values represent greater distance between two colors (averaged across all participants.). (C) Color map showing representational distance between brain patterns evoked by two color stimuli (averaged across all participants) in different regions.
